# Comparative Analysis of Cage Subsidence in Anterior Cervical Decompression and Fusion: Zero Profile Anchored Spacer (ROI-C) vs. Conventional Cage and Plate Construct

**DOI:** 10.3389/fsurg.2021.736680

**Published:** 2021-10-27

**Authors:** Zhe-yu Jin, Yun Teng, Hua-zheng Wang, Hui-lin Yang, Ying-jie Lu, Min-feng Gan

**Affiliations:** Department of Orthopedics, First Affiliated Hospital of Soochow University, Suzhou, China

**Keywords:** cage subsidence, anterior cervical decompression and fusion, over-distraction, multiple segments, zero-profile cages

## Abstract

**Background:** Anterior cervical discectomy and fusion (ACDF) has been widely performed to treat cervical generative diseases. Cage subsidence is a complication after ACDF. Although it is known that segmental kyphosis, acceleration of adjacent segmental disease, and restenosis may occur due to cages subsidence; however detailed research comparing zero-profile cages (ROI-C) and conventional plate and cage construct (CPC) on cage subsidence has been lacking.

**Objective:** The objectives of this study was to compare the rate of postoperative cage subsidence between zero profile anchored spacer (ROI-C) and conventional cage and plate construct (CPC) and investigate the risk factors associated with cage subsidence following ACDF.

**Methods:** Seventy-four patients with ACDF who received either ROI-C or CPC treatment from October 2013 to August 2018 were included in this retrospective cohort study. Clinical and radiological outcomes and the incidence of cage subsidence at final follow up-were compared between groups. All patients were further categorized into the cage subsidence (CS) and non-cage subsidence (NCS) groups for subgroup analysis.

**Results:** The overall subsidence rate was higher in the ROI-C group than in the CPC group (66.67 vs. 38.46%, *P* = 0.006). The incidence of cage subsidence was significantly different between groups for multiple-segment surgeries (75 vs. 34.6%, *P* = 0.003), but not for single-segment surgeries (54.55 vs. 42.30%, *P* = 0.563). Male sex, operation in multiple segments, using an ROI-C, and over-distraction increased the risk of subsidence. Clinical outcomes and fusion rates were not affected by cage subsidence.

**Conclusion:** ROI-C use resulted in a higher subsidence rate than CPC use in multi-segment ACDF procedures. The male sex, the use of ROI-C, operation in multiple segments, and over-distraction were the most significant factors associated with an increase in the risk of cage subsidence.

## Introduction

Anterior cervical decompression and fusion (ACDF) has been widely used as a surgical treatment method for cervical disc degenerative diseases since it was first developed by Smith and Robinson in 1958 (1). Augmentation through the use of anterior cervical plating provides immediate stabilization and the preservation of cervical alignment, preventing graft dislodgment and enhancing fusion rates. However, the implantation of anterior plating has also been associated with complications, including postoperative dysphagia, soft tissue damage, and hardware failure (2–4). Zero-profile anchored spacers (ROI-Cs) have become popular due to reduced damage to soft-tissues, lower blood loss, and the avoidance of hardware-related complications compared with traditional cage and plate constructs (CPCs) (5–7). Moreover, after the insertion of the anchors, ROI-Cs provide immediate stability, facilitating fusion (8). Cage subsidence is a common complication following ACDF and can result in the loss of disc height, disrupting the sagittal alignment of the spine, preventing solid fusion, and introducing restenosis of the foramina (9, 10). However, the impacts of cage subsidence on clinical outcomes remain controversial for the cervical spine (11). Several factors have been proposed to contribute to cage subsidence, including aggressive endplate preparation, osteoporosis, differences in treatment levels, cage size, and cage material (11–13). However, data comparing ROI-C cages and CPCs regarding cage subsidence are scarce.

Thus, the purposes of this study were (1) to retrospectively evaluate the clinical and radiological outcomes of ACDF treatments for cervical disc degenerative disease (CDDD) using ROI-Cs compared with CPC fixation, with a focus on cage subsidence; and (2) to identify the preoperative and perioperative risk factors associated with cage subsidence and determine the impact of subsidence on clinical and radiological outcomes.

## Materials and Methods

### Patient Population

This study was conducted as a retrospective analysis of 85 patients with one to three levels of CDDD who underwent ACDF from October 2013 to August 2018. The study was approved by the Medical Ethics Committee of the First Affiliated Hospital of Soochow University. Informed written consent was obtained from all included patients prior to surgery. The study inclusion criteria were as follows: (1) the clinical presentation of myelopathy or radiculopathy; (2) spinal cord or nerve root compression observed on recent magnetic resonance imaging (MRI); and (3) the failure of conservative treatment after a minimum of 6 months. The exclusion criteria were: (1) operations at the C2–3 or C7–T1 disc levels; (2) severe cervical instability, developmental stenosis, or the ossification of the posterior longitudinal ligament; (3) previous medical records of cervical surgery, trauma, metabolic diseases, infection, or tumor; and (4) follow-up less than 12 months. The following data were collected from patients' perioperative, surgical, and discharge records: demographic characteristics, surgical procedure, intraoperative blood loss, length of hospital stay. Follow-up clinical notes (postoperatively at 1 month and final follow-up) were reviewed to evaluate postoperative changes in clinical and radiographical outcomes.

### Surgical Method

All surgeries were performed by the same surgeon in this study. All surgical procedures were performed as previously described by our orthopedic center (3, 4). After general anesthesia, with the patient placed in supine position, the classic Robinson and Cloward anterior cervical approach and technique were used. Extensive decompression was performed, including the removal of osteophytes, herniated discs and posterior longitudinal ligament as indicated to achieve sufficient decompression of the spinal cords and nerve roots. The cartilage endplates were abraded carefully, and the bony endplates were preserved to prevent possible subsidence. No allograft was used. The choice of implant was according to surgeon's preference. Stand-alone PEEK cages were inserted into the disc space along with anterior cervical plates immobilized by self- tapping screws in the CPC group. For ROI-C group (ROI-C, LDR, Troyes, France), after insertion of a trial cage to confirm intraoperative stability, a ROI-C cage sized properly, and packed with autologs cancellous bone was then placed in the disc space using an impactor. Two anchoring chips were placed into the upper and lower vertebra under fluoroscopic guidance. Postoperatively, all patients were encouraged to exercise around their bedsides with the assistance of a semi-rigid neck collar 24 hours after surgery. Patients were strongly advised to refrain from excessive cervical movements for a minimum of 3 months after surgery.

### Clinical Evaluation

The modified Japanese Orthopedic Association (JOA) scoring system was used to assess preoperative and postoperative functional status. The Neck Disability Index (NDI) scoring system was used to determine disability caused by neck pain during daily life.

### Radiologic Assessment

**Figure 1:**
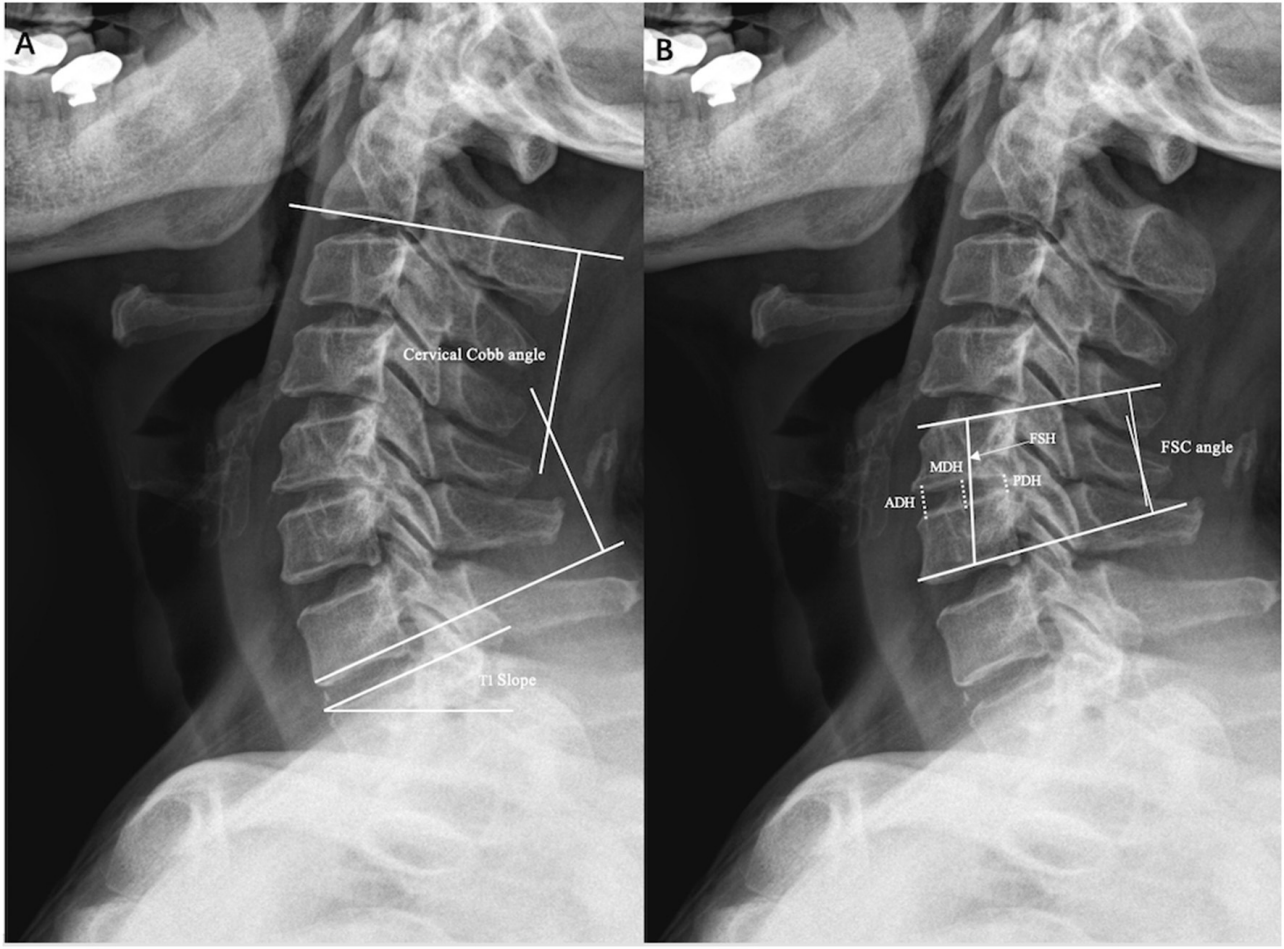
Illustrations of radiographic measurements. **(A)** Cervical cobb angle, T1 slope. **(B)** Fused segment cobb angle (FSC), anterior disc height (ADH), midline disc height (MDH), posterior disc height (PDH), and fused segment height (FSH).

The radiographic outcome was evaluated preoperatively and at each follow up time point. The Cobb angle of the cervical C2–C7 (CA) vertebrae was defined as the angle between the tangent lines of the lower C2 vertebral body endplates and the upper C7 vertebral body endplates. The T1 slope was measured as the angle formed between a horizontal line and the T1 upper endplate. If the T1 slope was not visible due to anatomical interference, the upper C7 slope was used instead (14) (Figure 1A). The fused segment Cobb angle (FSC) was defined as the Cobb angle that was formed by the fusion levels, as measured from the upper endplate of the upper vertebral body and the lower endplate of the lower vertebral body. The mean disc height (mDH) was evaluated as the mean value of the anterior disc height (ADH), the midline disc height (MDH), and the posterior disc height (PDH). The fusion segment height (FSH) was assessed as the distance from the midpoint of the upper endplate of the upper vertebral body of the fused segment to the midpoint of the lower endplate of the lower vertebral body (Figure 1B). Adjacent segment degeneration (ASD) was defined as new osteophyte formations or the enlargement of existing osteophytes, new disc space narrowing, or segmental instability visible on plain film radiographs, or any decrease in disc signal intensity or intervertebral herniation at adjacent segments observed on T2-weighted MRI (15, 16). The postoperative fusion was defined based on the assessment of the following features: (1) trabecular bridging across the bone-graft interface, (2) the absence of radiolucent gaps between the graft and the vertebral endplate, and (3) changes of less than 2 mm in the interspinous distance of the fused segments, assessed on lateral flexion-extension radiographs (17). Subsidence was defined as a greater than 2 mm reduction in mDH at the final follow-up compared with measurements taken at 1 month postoperatively.

All patients were further divided into a cage subsidence group (CS group) and a non-cage subsidence group (NCS group) to examine the risk factors associated with the incidence of postoperative cage subsidence. The factors assessed in this analysis included age, sex, the use of ROI-C cage, the number of operated levels (single vs. multiple), the affected levels (C3–C5 vs. C5–C7), preoperative cervical Cobb angle (CA), postoperative CA, change in CA (ΔCA = postoperative CA-preoperative CA), and change in mDH (ΔmDH = postoperative mDH-preoperative mDH).

### Statistical Analysis

All statistical analyses were performed using SPSS 24.0 software (SPSS Inc., Chicago, IL). All continuous variables were compared between groups using the independent *t*-test. All categorical variables are expressed as the number and percentage and were compared using the Chi-square or Fisher's exact test. To adjust for confounding variables, we performed a multivariate logistic regression analysis of the risk factors associated with subsidence that exhibited significance. A *P* < 0.05 was considered significant.

## Results

### Study Population

Ultimately, 74 patients (36 men and 38 women) were considered eligible for enrollment in this study. The cohort was first divided into two subgroups based on the types of implants received. The CPC group included 36 patients that received conventional polyetheretherketone (PEEK) cages and an anterior titanium plate, whereas the ROI-C group included 38 patients who underwent fusion utilizing zero-profile anchored spacers.

In the CPC group, the mean age and follow-up time were 49.7 ± 10.9 years (range: 32–73 years) and 28.06 ± 13.09 months (range: 12.07–56.83 months), respectively. In the ROI-C group, the mean age and follow-up time were 53.7 ± 9.98 years (range: 44–72 years) and 24.64 ± 9.786 (range: 13.57–23.80) months, respectively. The patient groups that received ROI-C and CPC spacers were closely matched in terms of patient number, age, sex, height, BMI, indications for surgery, and the number of operated levels, with no significant differences in any of these variables (*P* > 0.05). The use of ROI-C spacers was associated with less estimated blood loss compared with the use of the CPC. The length of hospital stays was slightly longer for the CPC group than for the ROI-C group, but this difference was not significant (*P* > 0.05). Table 1 summarizes the perioperative and postoperative data.

**Table 1:** Summary of preoperative and operative details.

**Variables**	**CPC**	**ROI-C**	***P*-value**
Patients (n)	38	36	
Gender (male/female)	19/19	17/19	0.821
Age (yr)	49.7 ± 10.9	53.7 ± 9.98	0.110
Height (cm)	164.7 ± 7.57	164.6 ± 7.27	0.955
BMI	24.44 ± 3.149	24.22 ± 3.163	0.772
Diagnosis (n)			
Radiculopathy	9	15	0.815
Myelopathy	22	19	0.137
Combined symptoms	7	2	0.153
Number of operated levels	52	54	0.623
One-level	26	22	
Two-level	10	10	
Three-level	2	4	
Operation time (min)	149.29 ± 47.80	144.78 ± 60.84	0.727
Estimated blood loss (mL)	107.37 ± 46.97	57.64 ± 36.10	<0.001^*^
Hospital stay, days	8.68 ± 5.57	7.69 ± 3.96	0.390
Follow-up period, months	28.06 ± 13.09	24.64 ± 9.79	0.209

* *Statistically significant difference (P < 0.05)*.

### Clinical and Radiological Outcomes

The JOA and NDI scores improved significantly compared with baseline data for both groups (*P* < 0.01). No significant differences were observed in the JOA and NDI scores between the two groups at the final follow up (*P* > 0.05, Table 2). The T1 slope values showed no significant differences between two groups at each follow-up time point (*P* > 0.05). The mDH and FSH values after surgery for both groups increased significantly (*P* < 0.01), with no significant differences between the two groups at each follow-up time point, indicating the restoration of disc height (*P* > 0.05). At the final follow-up, reductions in cervical Cobb angle, mDH, and FSH value were observed compared with the postoperative values for both groups. These values for both groups were well maintained postoperatively at the final follow-up. Table 3 shows the radiologial outcomes.

**Table 2:** Clinical outcomes between ROI-C and CPC group.

**Variables**	**ROI-C**	**CPC**	***P*-value**
JOA Scores			
Preop	11 ± 0.93	11.2 ± 0.8	0.500
Postop 1 M	15.58 ± 0.63	15.67 ± 0.67	0.569
Final FU	16.8 ± 0.43	16.9 ± 0.35	0.290
NDI scores			
Preop	40.88 ± 6.57	37.76 ± 6.011	0.039^*^
Postop 1 M	16 ± 4.34	16.4 ± 4.82	0.740
Final FU	8.95 ± 4.44	7.5 ± 3.69	0.140

* *Statistically significant difference (P < 0.05)*.

**Table 3:** Radiographic outcomes between ROI-C group and CPC group.

**Variable**	**Roi-C (36)**	**CPC (38)**	***P*-value**
C2-C7 CA			
Preop	13 ± 8.7	12 ± 9.2	0.700
Postop 1 M	15 ± 8.1	14 ± 8.4	0.700
Final FU	13.6 ± 9.07	13.2 ± 8.72	0.86
T1 Slope			
Preop	21.62 ± 7.12	21.45 ± 8.59	0.929
Postop 1 M	22.83 ± 7.49	22.18 ± 6.92	0.705
Final FU	21.5 ± 7.76	22.8 ± 7.07	0.470
mDH (mm)			
Preop	5.26 ± 0.81	5.43 ± 1.06	0.360
Postop 1 M	8.62 ± 1.51	8.41 ± 1.48	0.460
Final FU	6.8 ± 4.05	6.57 ± 1.25	0.703
FSH			
Preop	31.35 ± 4.53	30.74 ± 3.65	0.452
Postop 1 M	37.41 ± 6.30	37.27 ± 5.745	0.907
Final FU	33.77 ± 10.37	33.10 ± 9.00	0.728
Subsidence, n (%)			
2 mm	36/54 (66.67%)	20/52 (38.46%)	0.006^*^
Single level	12/22 (54.55%)	11/26 (42.30%)	0.563
Multiple level	24/32 (75.00%)	9/26 (34.62%)	0.003^*^
Fusion rate			
Postop 3 M	31/36 (86.1%)	30/38 (78.95%)	0.545
Final FU	35/36 (97.2)	35/38 (92.1%)	0.615
ASD	5/36 (13.9%)	9/38 (23.7%)	0.377

* *Statistically significant difference (P < 0.05)*.

Radiological evidence of ASD was identified in nine cases (23.7%) in the ROI-C group and 5 cases (13.9%) in the CPC group. The fusion rates at the final follow-up for the CPC group and the ROI-C group were 92.1 and 97%, respectively. No significant difference was observed between either the ASD rates or the fusion rates between the two groups (*P* > 0.05).

### Cage Subsidence

Subsidence was more frequently observed in the ROI-C group (66.7%) than in the CPC group (38.5%, *P* = 0.037). Cage subsidence was not observed at immediate postoperatvie radiographs in both groups. At the final follow-up, the overall rate of cage subsidence was 62.26% (66/106 levels), occurring in 42 patients (55.26%, Table 3). Among single-level ACDFs, the occurrence of subsidence was not significantly different between the two groups. However, among multiple-level ACDFs, the subsidence rate was higher for the ROI-C group than for the CPC group (75.00 vs. 34.62%, *P* = 0.003).

### Subgroup Analysis

#### Clinical and Radiological Outcomes

At the final follow-up, the JOA scores for the NCS group and the CS group were 16.219 ± 1.157 and 16.381 ± 1.103, respectively. The NDI scores were 8.938 ± 4.250 and 7.143 ± 4.194, respectively. No significant difference was observed for either value between the two groups (*P* > 0.05). In the CS group, 73 levels (96.05%) achieved fusion. In the NCS group, 29 levels (96.67%) achieved fusion, with no significant difference between groups (*P* > 0.05). ASD rates also showed no significant difference between groups (*P* > 0.05).

**Figure 2:**
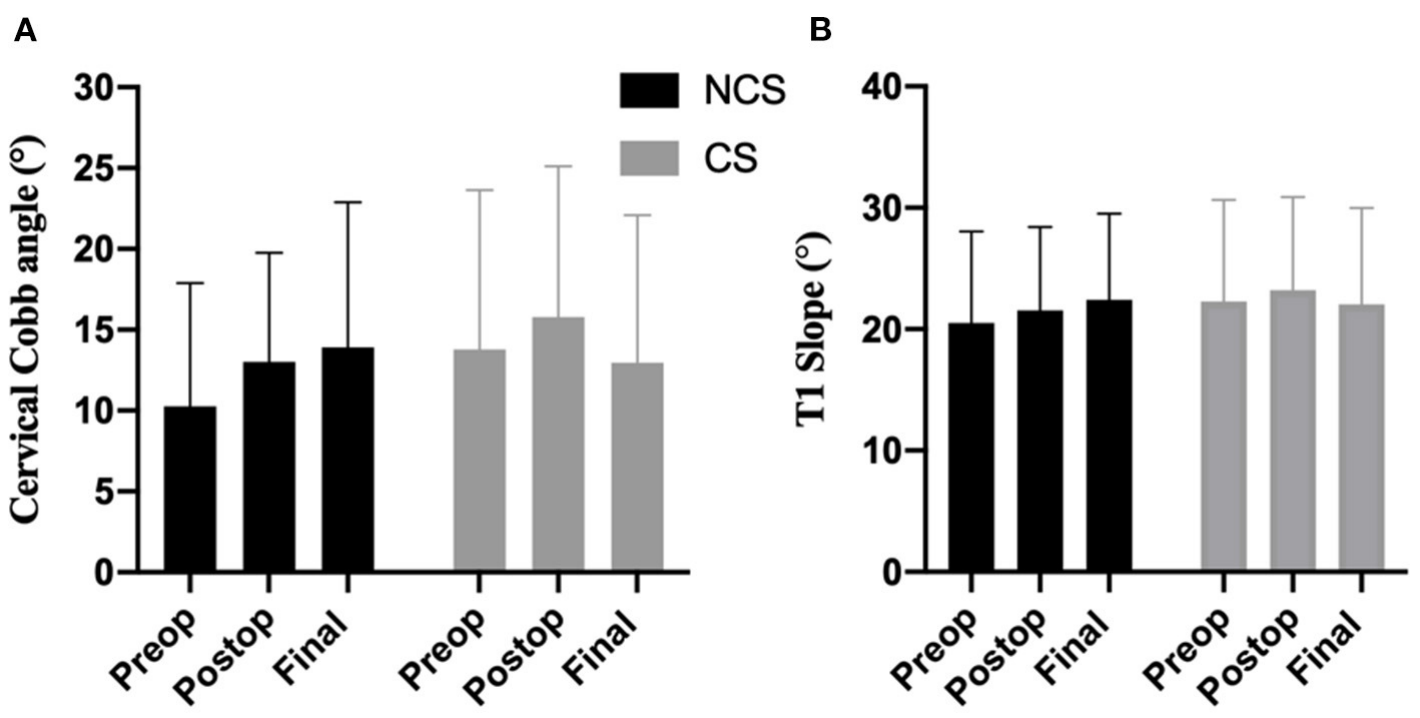
The mean cervical Cobb angle **(A)** and T1 slope **(B)** values in CS (cage subsidence) and NCS (non-cage subsidence) groups.

No significant difference was found in the mean Cobb angle and T1 slope values between the CS and NCS groups at any time follow up point. In the CS group, the mean Cobb angle decreased at the final follow-up compared with postoperative value (15.79 ± 9.33 vs. 12.95 ± 9.14); whereas a small increase in the mean Cobb angle after surgery was observed for the NCS group (13.00 ± 6.77 vs. 13.91 ± 8.98); however, these differences were not significant for either group (*P* > 0.05, Figure 2). The ΔmDH in the CS group was significantly higher than that of the NCS group (3.849 ± 1.586 mm vs. 0.422 ± 1.311 mm, *P* < 0.001). The ΔFSH in the CS group (11.57 ± 8.827) was also higher than that of the NCS group (4.61 ± 4.392, *P* < 0.001). These results indicated that the cervical disc spaces were excessively distracted after the insertion of cages in the CS group compared with that of the NCS group.

**Figure 3:**
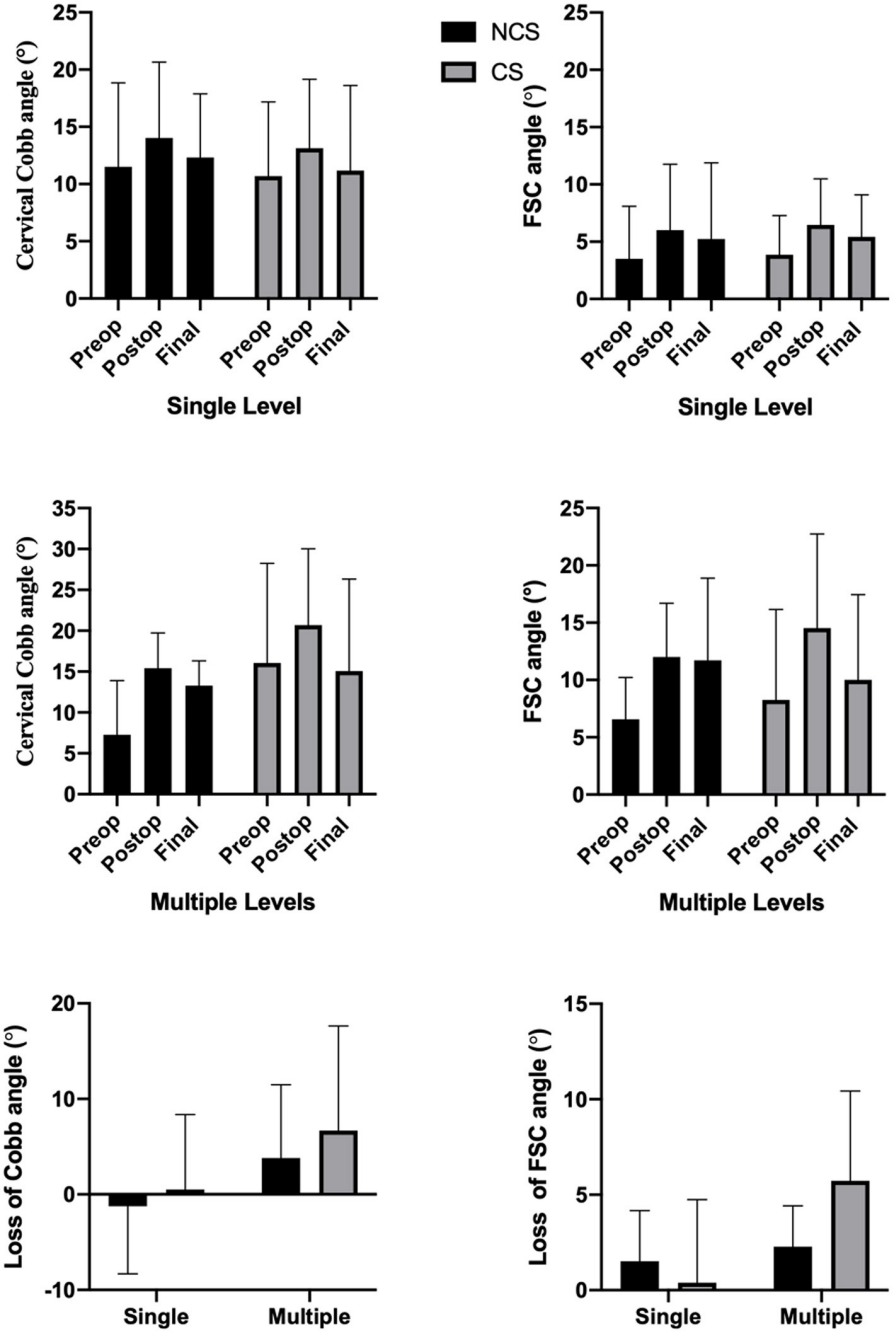
The mean cervical Cobb angle, FSC angle, the loss of mean Cobb angle, and FSC angle in single level and multiple levels between CS and NCS groups.

To compare the effect of cage subsidence on local and general curvature, groups were further divided into single level and multiple levels (Figure 3). The loss of both FSC angle (6.68 ± 10.95 vs. 0.52 ± 7.8) and cervical Cobb angle (3.82 ± 7.67 vs. −1.24 ± 7.07) were more pronounced in multiple levels with cage subsidence, but not in single level ACDFs. However, both values failed to reach statistically significant difference (*P* > 0.05).

## Risk Factors of Subsidence

After univariate analysis, we identified the following factors as being associated with an increase in the risk of cage subsidence: male sex (*P* < 0.001), the use of ROI-C cage (*P* = 0.007), operation at multiple levels (*P* = 0.024), and ΔmDH (*P* < 0.001, Table 4). Multiple logistic regression was performed by analyzing these variables. The results revealed that the risk of cage subsidence was significantly associated with the male sex (OR = 16.767; *P* < 0.001) and the use of ROI-C cage (OR = 5.389; *P* < 0.001, Table 5).

**Table 4:** Univariate analysis of clinical and radiological factors between groups.

**Variables (n)**	**CS (42 patients,56 cages)**	**NCS (32 patients, 50 cages)**	***P-value***
Age, years	53.60 ± 11.19	49.03 ± 9.707	0.070
Gender (male/female)	30/12	6/26	<0.001^*^
BMI	24.43 ± 2.93	24.20 ± 3.53	0.764
**Surgical methods**			
CPC	17 (44.74%)	21(55.26%)	0.038^*^
ROI-C	25 (69.44%)	11(30.56%)	
**Number of operated levels**			0.037^*^
1	23 (47.92%)	25 (52.08%)	
≥2	19 (73.08%)	7 (26.92%)	
**Subsidence levels**			0.222
C3-5	28	12	
C5-7	38	28	
Pre-op CA	13.79 ± 9.84	10.25 ± 7.65	0.097
Post-op CA	15.79 ± 9.33	13.00 ± 6.77	0.158
ΔCA	2.000 ± 10.44	2.750 ± 7.348	0.730
ΔmDH	3.849 ± 1.586	2.422 ± 1.311	<0.001^*^

* *Statistically significant difference (P < 0.05)*.

**Table 5:** Multivariate analysis of the risk factors.

**Variables**	**Odds Ratio (95%CI)**	***P*-value**
Gender (Male vs. Female)	16.767	<0.001
Operation method (CPC vs. ROI-C)	5.389	0.012
Number of discectomies (1 vs. ≥2)	3.183	0.084

## Discussion

The patient-reported outcomes in our current study results were consistent with those that have previously been published in the literature for ACDF when comparing zero-profile stand-alone locking screws and CPC (5–7). All neurological symptoms were relieved due to sufficient decompression, and no significant differences were observed in either the JOA or NDI scores between the groups at the final follow-up. However, in this study, we found that the occurrence of cage subsidence in patients using the ROI-C cage (19/26, 73.08%) was significantly higher than that for the CPC group (7/26, 26.92%) when the treatment involved multiple-level discectomies, although no significant difference was found for single-level surgeries.

After surgery, the occurrence of cage subsidence was frequently observed in ACDF surgeries, with a mean incidence of 21.1%, ranging from 0 to 83% (18). Earlier studies also reported a higher rate of cage subsidence when using ROI-C compared with CPC for the treatment of multiple-level ACDFs. In a meta-analysis conducted by Lu et al. (19) no significant differences were found between the zero-profile self-locking standalone cages (SLSA) and CPC group after performing single-segment ACDF, whereas increased subsidence was demonstrated in the zero-profile group for multi-segment ACDFs. According to Chen et al. (20) for the treatment of three-level cervical degenerative spondylopathy, cage subsidence of greater than 3 mm was observed in 14/28 patients in the SLSA group compared with 5/26 patients cage and plate fixation group at the final follow-up (*P* = 0.043). Similarly, Zhu et al. (21) reported 17/90 (18.8%) patients in the SLSA group experienced subsidence compared with 8/96 (8.3%) patients in the CPC group for three-level ACDF (*P* > 0.05). From a biomechanical view, the loading pressure that is directly delivered to endplate/cage interfaces can be shared by anterior metal plating. In multi-segment ACDFs, this effect can be more pronounced relative to that in single-level discectomies, which can attenuate the risk of cage subsidence, resulting in a higher incidence of cage subsidence when using anchored cages.

Surprisingly, we noticed a sex difference in the occurrence of cage subsidence, with significantly higher rates observed in men than in women. At our spine center, patients are recommended to wear cervical collars for at least 1 month postoperatively and to refrain from excessive movements. One possible explanation is the early removal of the cervical collar after surgery and a more aggressive range of motion among men compared with women. Indeed, aggressive cervical movement in the early postoperative period can cause larger axial and rotational stress upon the interbody/cage interface, which can result in cage subsidence before solid fusion. However, this study did not aim to collect data regarding the timing of cervical collar removal or the impact of cervical collar removal on the clinical and radiological outcomes after surgery; therefore, additional research is necessary.

Cages are inserted to maintain the clinical efficacy of decompression. However, overly distracted disc space can increase the risk of cage subsidence. Yang et al. (22) confirmed that a larger anterior intraoperative distraction increased the risk of cage subsidence and recommended that interbody distraction be performed before anterior longitudinal ligament resection. Similarly, Yamagata et al. (23) demonstrated that using a titanium cage for ACDF with a size of 6.5 or 7.5 mm had a higher rate of subsidence than when using a titanium cage for an ACDF with a size of 4.5 or 5.5 mm. According to an *in vitro* biomechanical study performed by Truumees et al. (24) the insertion of larger grafts results in higher distractive forces and increases the subsequent compressive forces delivered to the endplate-cage interface. These authors further proved that distractive force and the subsequent compressive forces were strongly correlated in an *in vivo* ACDF model (25). After the restoration of disc height via cage insertion, the increase in disc height causes the surrounding ligaments and muscles to absorb and resist distraction forces, contributing to the immediate compression of the graft. In the present study, the ΔmDH and ΔFSH in the CS group were significantly higher than those in the NCS group. The results of our study, combined with those of other studies, suggested that excessive distraction should be avoided to reduce the risk of potential cage subsidence in ACDF surgery.

**Figure 4:**
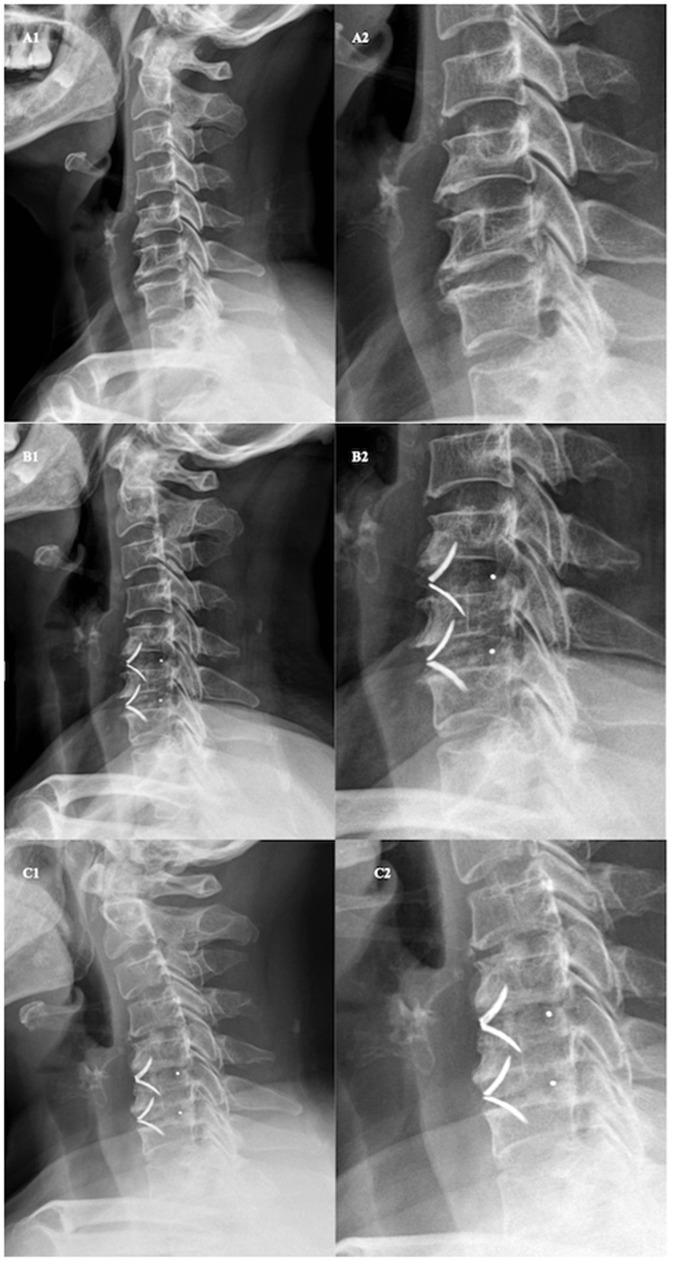
Demonstration of cage subsidence after ACDF and its influence on local and general cervical lordosis. Lateral radiograph **(A1)** and enhanced view **(A2)** before anterior cervical discectomy and fusion with ROI-C cage. Lateral radiograph **(B1)** and enhanced view **(B2)** one month after ACDF with ROI-C cage. Lateral radiograph **(C1)** and enhanced view **(C2)** one year after ACDF with ROI-C cage. Fused segment cobb angle were 0°, 10°, and, 0° and Cervical cobb angle were 2°, 5°, and −15° before operation, 1-month post-op, and 1-year post-op respectively.

Cage subsidence has been reported to cause loss of fused segment height, further leading to disruption of cervical stability (13, 20). Similarly, we found that the loss of cervical lordosis and fusion segment cobb angle were more pronounced in CS group (Figure 4). However, the impact of subsidence on loss of general and local lordosis was mainly observed in the treatment of multiple segments. Whereas, in single level surgeries, cage subsidence had little or no effect (Figure 3). This suggests that the increase in the number of fused segments would increase the effect of subsidence upon both local and general curvature. However, the overall clinical outcomes, fusion rates, and ASD rates were not associated with cage subsidence in our study. Previous studies have proposed that cage subsidence may represent an inherent process that occurs during the fusion of the bony endplates with the interbody cage, which includes the resorption and remodeling of the bone until rigid arthrodesis occurs (26, 27). Fujibayashi et al. (9) further divided cage subsidence into two types: a transient subsidence type demonstrates 1–3 mm subsidence without further change, whereas a progressive subsidence type results in nonunion. A systemic review by Noordhoek et al. (18) was unable to conclude that subsidence impacts clinical outcomes and fusion. Wu et al. (28) reported that cervical lordosis, rather than cage subsidence, had the most effect on long-term clinical and radiological outcomes. However, from a biomechanical standpoint, progressive subsidence is likely to result in the recompression of nerves after initial decompression. Surgeons should be highly aware of the risk factors for subsidence to avoid its occurrence. Previously reported risk factors and those identified in the present study include and are not limited to age, sex, bone density, endplate preparation, cage material and position, over-distraction, and multi-segment fusion (22, 29–31).

The limitations of the present study include its retrospective nature and the lack of randomization between procedures. Second, this study did not include osteoporosis as a risk factor that may influence cage subsidence. Third, although postoperative CT (93.2%) were taken for most patients, an inconsistency among the imaging techniques used for evaluation was present during follow-up, which may cause variations in radiographic measurements. Longer follow-up period and larger number of patients remains necessary to evaluate changes in subsidence over time and to determine its impact on clinical outcomes and cervical alignments.

## Conclusion

In our study, ACDF with ROI-C cage achieved comparable clinical outcomes and cervical stability compared with the use of a CPC. However, our study demonstrated that the occurrence of cage subsidence was considerably higher in the ROI-C group when multiple-level surgeries were performed compared with that in the CPC group. We further identified that male sex, the use of a ROI-C cage, multiple-level discectomies, and over-distraction were significant risk factors for cage subsidence. Despite no correlation between cage subsidence and clinical outcomes was observed in our study, the potential drawbacks of cage subsidence should be considered when using the ROI-C cage in multiple-level ACDFs.

## Data Availability Statement

The original contributions presented in the study are included in the article/supplementary material, further inquiries can be directed to the corresponding author/s.

## Ethics Statement

The studies involving human participants were reviewed and approved by Medical Ethics Committee of the First Affiliated Hospital of Soochow University (IRB#2021-119). The patients/participants provided their written informed consent to participate in this study. Written informed consent was obtained from the individual(s) for the publication of any potentially identifiable images or data included in this article.

## Author Contributions

M-fG, Y-jL, and H-lY contributed to the study concept and design. Z-yJ, YT, and H-zW contributed to the acquisition of data and analysis and interpretation of data. Z-yJ contributed to the drafting of the manuscript. All authors have read and approved the final manuscript.

## Conflict of Interest

The authors declare that the research was conducted in the absence of any commercial or financial relationships that could be construed as a potential conflict of interest.

## Publisher's Note

All claims expressed in this article are solely those of the authors and do not necessarily represent those of their affiliated organizations, or those of the publisher, the editors and the reviewers. Any product that may be evaluated in this article, or claim that may be made by its manufacturer, is not guaranteed or endorsed by the publisher.

## References

[1] Smith GW Robinson RA. The treatment of certain cervical-spine disorders by anterior removal of the intervertebral disc and interbody fusion. J Bone Joint Surg Am. (1958) 40:607–24. 10.2106/00004623-195840030-00009 13539086

[2] Fountas KN Kapsalaki EZ Nikolakakos LG Smisson HF Johnston KW Grigorian AA. Anterior cervical discectomy and fusion associated complications. Spine (Phila Pa 1976). (2007) 32:2310–7. 10.1097/BRS.0b013e318154c57e 17906571

[3] Gazzeri R Tamorri M Faiola A Gazzeri G. Delayed migration of a screw into the gastrointestinal tract after anterior cervical spine plating. Spine (Phila Pa 1976). (2008) 33: E268–271. 10.1097/BRS.0b013e31816b8831 18404097

[4] Amelot A Bouazza S George B Orabi M Bresson D. Anterior extrusion of fusion cage in posttraumatic cervical disk disease. J Neurol Surg A Cent Eur Neurosurg. (2015) 76:168–71. 10.1055/s-0034-1389095 25306206

[5] Grasso G Giambartino F Tomasello G Iacopino G. Anterior cervical discectomy and fusion with ROI-C peek cage: cervical alignment and patient outcomes. Eur Spine J. (2014) 23 (Suppl. 6):650–7. 10.1007/s00586-014-3553-y 25200146

[6] Wang Z Jiang W Li X Wang H Shi J Chen J. The application of zero-profile anchored spacer in anterior cervical discectomy and fusion. Eur Spine J. (2015) 24:148–54. 10.1007/s00586-014-3628-9 25337859

[7] Lu Y Bao W Wang Z Zhou F Zou J Jiang W. Comparison of the clinical effects of zero-profile anchored spacer (ROI-C) and conventional cage-plate construct for the treatment of noncontiguous bilevel of cervical degenerative disc disease (CDDD): a minimum 2-year follow-up. Medicine (Baltimore). (2018) 97:e9808. 10.1097/MD.0000000000009808 29384883 PMC5805455

[8] Bucci MN Oh D Cowan RS Davis RJ Jackson RJ Tyndall DS. The ROI-C zero-profile anchored spacer for anterior cervical discectomy and fusion: biomechanical profile and clinical outcomes. Med Devices. (2017) 10:61–9. 10.2147/MDER.S127133 28458586 PMC5403002

[9] Fujibayashi S Neo M Nakamura T. Stand-alone interbody cage versus anterior cervical plate for treatment of cervical disc herniation: sequential changes in cage subsidence. J Clin Neurosci. (2008) 15:1017–22. 10.1016/j.jocn.2007.05.011 18653347

[10] Kast E Derakhshani S Bothmann M Oberle J. Subsidence after anterior cervical inter-body fusion. A randomized prospective clinical trial. Neurosurg Rev. (2009) 32:207–14; discussion 214. 10.1007/s10143-008-0168-y 18797946

[11] Igarashi H Hoshino M Omori K Matsuzaki H Nemoto Y Tsuruta T. Factors Influencing Interbody Cage Subsidence Following Anterior Cervical Discectomy and Fusion. Clin Spine Surg. (2019) 32:297–302. 10.1097/BSD.0000000000000843 31169615

[12] Kao TH Wu CH Chou YC Chen HT Chen WH Tsou HK. Risk factors for subsidence in anterior cervical fusion with stand-alone polyetheretherketone (PEEK) cages: a review of 82 cases and 182 levels. Arch Orthop Trauma Surg. (2014) 134:1343–51. 10.1007/s00402-014-2047-z 25099076 PMC4168225

[13] Lee YS Kim YB Park SW. Risk factors for postoperative subsidence of single-level anterior cervical discectomy and fusion: the significance of the preoperative cervical alignment. Spine (Phila Pa 1976). (2014) 39:1280–7. 10.1097/BRS.0000000000000400 24827519

[14] Tamai K Buser Z Paholpak P Sessumpun K Nakamura H Wang JC. Can C7 Slope Substitute the T1 slope?: an analysis using cervical radiographs and kinematic MRIs. Spine (Phila Pa 1976). (2018) 43:520–525. 10.1097/BRS.0000000000002371 28767624

[15] Miyazaki M Hong SW Yoon SH Morishita Y Wang JC. Reliability of a magnetic resonance imaging-based grading system for cervical intervertebral disc degeneration. J Spinal Disord Tech. (2008) 21:288–92. 10.1097/BSD.0b013e31813c0e59 18525490

[16] Song KJ Choi BW Jeon TS Lee KB Chang H. Adjacent segment degenerative disease: is it due to disease progression or a fusion-associated phenomenon? Comparison between segments adjacent to the fused and non-fused segments. Eur Spine J. (2011) 20:1940–5. 10.1007/s00586-011-1864-9 21656051 PMC3207329

[17] Pitzen TR Chrobok J Stulik J Ruffing S Drumm J Sova L. Implant complications, fusion, loss of lordosis, and outcome after anterior cervical plating with dynamic or rigid plates: two-year results of a multi-centric, randomized, controlled study. Spine (Phila Pa 1976). (2009) 34:641–6. 10.1097/BRS.0b013e318198ce10 19287352

[18] Noordhoek I Koning MT Jacobs WCH Vleggeert-Lankamp CLA. Incidence and clinical relevance of cage subsidence in anterior cervical discectomy and fusion: a systematic review. Acta Neurochir (Wien). (2018) 160:873–80. 10.1007/s00701-018-3490-3 29468440 PMC5859059

[19] Lu Y Fang Y Shen X Lu D Zhou L Gan M. Does zero-profile anchored cage accompanied by a higher postoperative subsidence compared with cage-plate construct? A meta-analysis. J Orthop Surg Res. (2020) 15:189. 10.1186/s13018-020-01711-9 32448320 PMC7247200

[20] Chen Y Lu G Wang B Li L Kuang L. A comparison of anterior cervical discectomy and fusion (ACDF) using self-locking stand-alone polyetheretherketone (PEEK) cage with ACDF using cage and plate in the treatment of three-level cervical degenerative spondylopathy: a retrospective study with 2-year follow-up. Eur Spine J. (2016) 25:2255–62. 10.1007/s00586-016-4391-x 26906171

[21] Zhu D Zhang D Liu B Li C Zhu J. Can self-locking cages offer the same clinical outcomes as anterior cage-with-plate fixation for 3-level anterior cervical discectomy and fusion (ACDF) in mid-term follow-up? Med Sci Monit. (2019) 25:547–57. 10.12659/MSM.911234 30659165 PMC6347916

[22] Yang JJ Yu CH Chang BS Yeom JS Lee JH Lee CK. Subsidence and nonunion after anterior cervical interbody fusion using a stand-alone polyetheretherketone (PEEK) cage. Clin Orthop Surg. (2011) 3:16–23. 10.4055/cios.2011.3.1.16 21369474 PMC3042165

[23] Yamagata T Takami T Uda T Ikeda H Nagata T Sakamoto S. Outcomes of contemporary use of rectangular titanium stand-alone cages in anterior cervical discectomy and fusion: cage subsidence and cervical alignment. J Clin Neurosci. (2012) 19:1673–8. 10.1016/j.jocn.2011.11.043 23084624

[24] Truumees E Demetropoulos CK Yang KH Herkowitz HN. Effects of disc height and distractive forces on graft compression in an anterior cervical discectomy model. Spine (Phila Pa 1976). (2002) 27:2441–5. 10.1097/00007632-200211150-00005 12435972

[25] Francke EI Demetropoulos CK Agabegi SS Truumees E Herkowitz HN. Distractive force relative to initial graft compression in an *in vivo* anterior cervical discectomy and fusion model. Spine (Phila Pa 1976). (2010) 35:526–30. 10.1097/BRS.0b013e3181bb0e6e 20147873

[26] Borm W Seitz K. Use of cervical stand-alone cages. Eur Spine J. (2004) 13:474–475; author reply 476-477. 10.1007/s00586-004-0707-3 PMC3476589 15112077

[27] Zhang JD Poffyn B Sys G Uyttendaele D. Are stand-alone cages sufficient for anterior lumbar interbody fusion? Orthop Surg. (2012) 4:11–4. 10.1111/j.1757-7861.2011.00164.x 22290813 PMC6583365

[28] Wu WJ Jiang LS Liang Y Dai LY. Cage subsidence does not, but cervical lordosis improvement does affect the long-term results of anterior cervical fusion with stand-alone cage for degenerative cervical disc disease: a retrospective study. Eur Spine J. (2012) 21:1374–82. 10.1007/s00586-011-2131-9 22205113 PMC3389116

[29] Cheng CC Ordway NR Zhang X Lu YM Fang H Fayyazi AH. Loss of cervical endplate integrity following minimal surface preparation. Spine (Phila Pa 1976). (2007) 32:1852–5. 10.1097/BRS.0b013e31811ece5a 17762292

[30] Opsenak R Hanko M Snopko P Varga K Kolarovszki B. Subsidence of anchored cage after anterior cervical discectomy. Bratisl Lek Listy. (2019) 120:356–61. 10.4149/BLL_2019_058 31113198

[31] Jang HJ Chin DK Kim KH Park JY. Does graft position affect subsidence after anterior cervical discectomy and fusion? Global Spine J. (2020). 10.1177/2192568220963061. [Epub ahead of print]. 33043700 PMC9109557

